# Trends in hospitalization of preterm infants with intraventricular hemorrhage and hydrocephalus in the United States, 2000-2010

**DOI:** 10.1186/2045-8118-12-S1-O1

**Published:** 2015-09-18

**Authors:** Eisha Anne Christian, Diana Jin, Frank Attenello, Timothy Wen, Steven Cen, William J Mack, Mark D Krieger, J Gordon McComb

**Affiliations:** 1 University of Southern California, Los Angeles, CA, USA; 2 Children's Hospital Los Angeles, CA, USA

## Objective

Even with improved prenatal and neonatal care, intraventricular hemorrhage (IVH) occurs in approximately 25-30% of preterm infants, and a subset of these patients develop hydrocephalus. We aim to describe current trends in hospitalization of preterm infants with IVH and post-hemorrhagic hydrocephalus (PHH) using the Nationwide Inpatient Sample (NIS) and Kids Inpatient Database (KID).

## Methods

The KID and NIS databases were combined to generate data for the years 2000 – 2010. All neonatal discharges with ICD9-CM codes for preterm birth with IVH alone or with IVH and hydrocephalus were included.

## Results

There were 147,823 preterm neonates with IVH, and 9% of this group developed hydrocephalus during the same admission. Twenty-five percent and 28% of patients with Grades 3 and 4 IVH respectively developed hydrocephalus in comparison to 1% and 4% of patients with Grades 1 and 2 IVH. Thirty-eight percent of patients with PHH had permanent ventricular shunts inserted. Mortality rates were 4%, 10%, 18%, and 40% respectively for Grades 1-4 during initial hospitalization. Length of stay has been trending upward for both groups of IVH (49d in 2000, 56d in 2010) and PHH (59d in 2000, 70d in 2010). Average hospital cost per patient (inflation-adjusted) has also increased from $201,578 to $353,554 (IVH) and $260,077 to $495,697 (PHH) over 11 years.

## Conclusion

The number of admissions of neonates with IVH has increased despite a decrease in the number of preterm births. Rates of hydrocephalus and mortality correlated closely with IVH grade. Incidence of hydrocephalus in preterm infants with IVH remained stable between 8-10%. Over an 11-year period, there was a progressive increase in hospital cost and length of stay for preterm neonates with IVH and PHH with a concurrent increase in the proportion of patients with congenital cardiac anomalies.
